# Case Series: A case of familial thymomatous myasthenia gravis in a family of three male brothers

**DOI:** 10.1093/jscr/rjaa321

**Published:** 2020-09-26

**Authors:** Sheref A Elseidy, Ahmed Abd Allah Abd Alkader, Haitham Hassan Naserallah, Ahmed Khaled Awad

**Affiliations:** Department of Cardiovascular Diseases, Ain Shams University, Cairo, Egypt; Department of Rheumatology, Ain Shams University, Cairo, Egypt; Department of Cardiovascular Diseases, Ain Shams University, Cairo, Egypt; Department of Cardiovascular Diseases, Ain Shams University, Cairo, Egypt

**Keywords:** Myasthenia gravis, Thymoma, Familial thymomatous myasthenia gravis, Acetylcholine receptors antibodies, Sibling, Thymectomy

## Abstract

Myasthenia gravis (MG) is an autoimmune disease that occurs as a consequence of anti-acetylcholine (Ach) antibodies specifically targeting postsynaptic Ach receptors (AchR). This leads to the evolution of the classic symptoms of the disease, which range from mild symptoms of diplopia, muscle fatigue with repetitive movement up to severe affection of the respiratory muscle. The disease can occur as an isolated finding or co-exist with a concomitant thymic tumor or hyperplasia. Careful diagnosis is crucial for the development of the management plan. Nearly 10–15% of MG cases coexist with a thymic pathology and in these cases, surgical resection leads to the resolution of symptoms. Although thymomatous MG occurrence is non-heritable, its polygenic nature accounts for its rare familial variant. In this case, we report a family of three brothers with familial thymomatous MG who underwent thymectomy and improved after thymic surgical resection. Myasthenia gravis can occur as an isolated finding or as an association of thymic pathology. Careful discrimination between the two should be made for the elaboration of a management plan. Familial variant thymomatous myasthenia gravis is exceedingly rare. A familial survey is crucial for its management.

## INTRODUCTION

Myasthenia gravis (MG) is a rare, autoimmune neuromuscular junction disorder with 1/5000 prevalence rates. Myasthenia gravis involves specific muscle groups and presents with painless, fluctuating, fatigable weakness. With initial presentation of Ocular weakness with asymmetric ptosis and binocular diplopia, myasthenia gravis is less common to present with secluded oropharyngeal or limb weakness. The course is flexible, and most patients with initial ocular weakness develop bulbar or limb weakness within 3 years of initial symptom onset. Myasthenia gravis results from antibody-mediated, T cell-dependent immunologic attack on the endplate region of the postsynaptic membrane of the neuromuscular junction. Nearly 10–15% of MG patients present with thymic pathology—mostly thymoma. The role of the thymus in the pathophysiology of MG is still a matter of debate and whether thymectomy or immunosuppresive drugs is the most effective has long been a controversial matter, but recently, the studies showed the efficacy of thymectomy—when performed early- for long-term improvement of MG patients.

## CASE PRESENTATION

### Case 1

**Figure 1:**
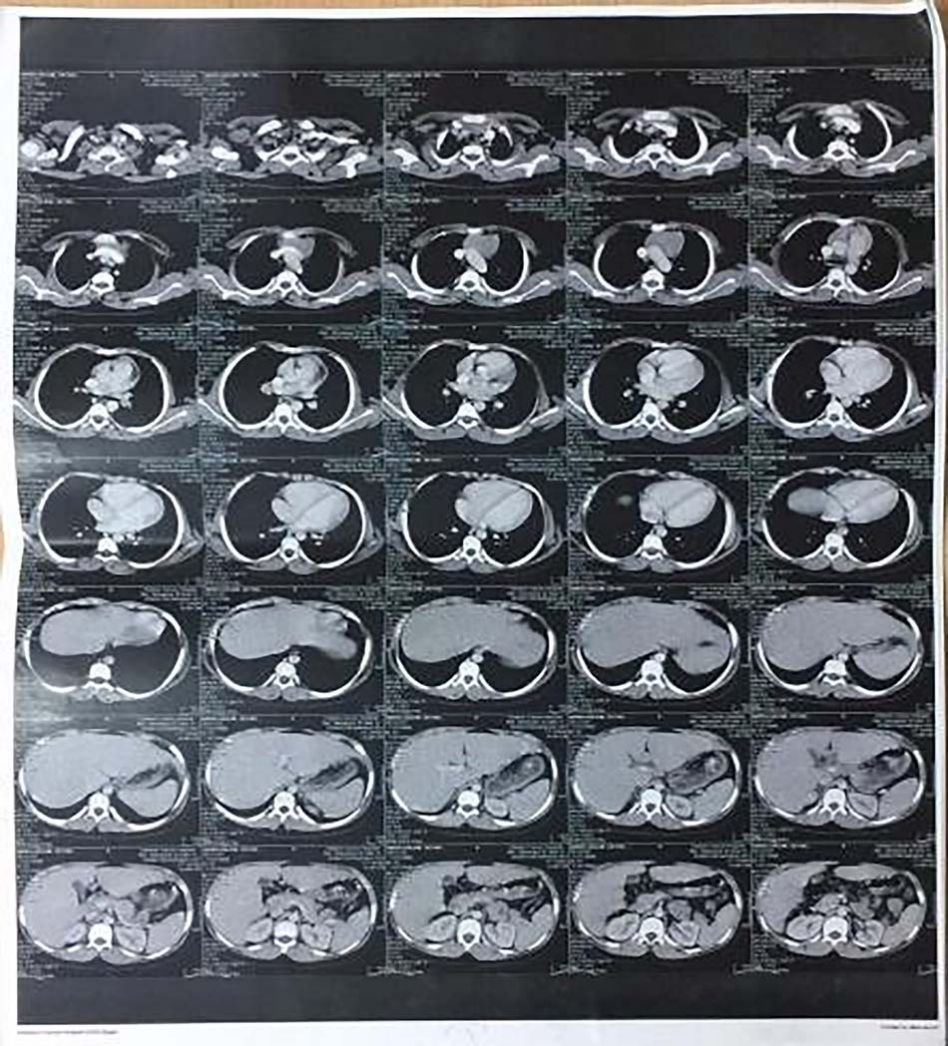
CT of regressed thymic homogenous tissue with clear lung fields; no pleural, pericardial effusion or evidence of enlarged mediastinal or hilar lymph node; the heart and great vessels were normal.

**Figure 2:**
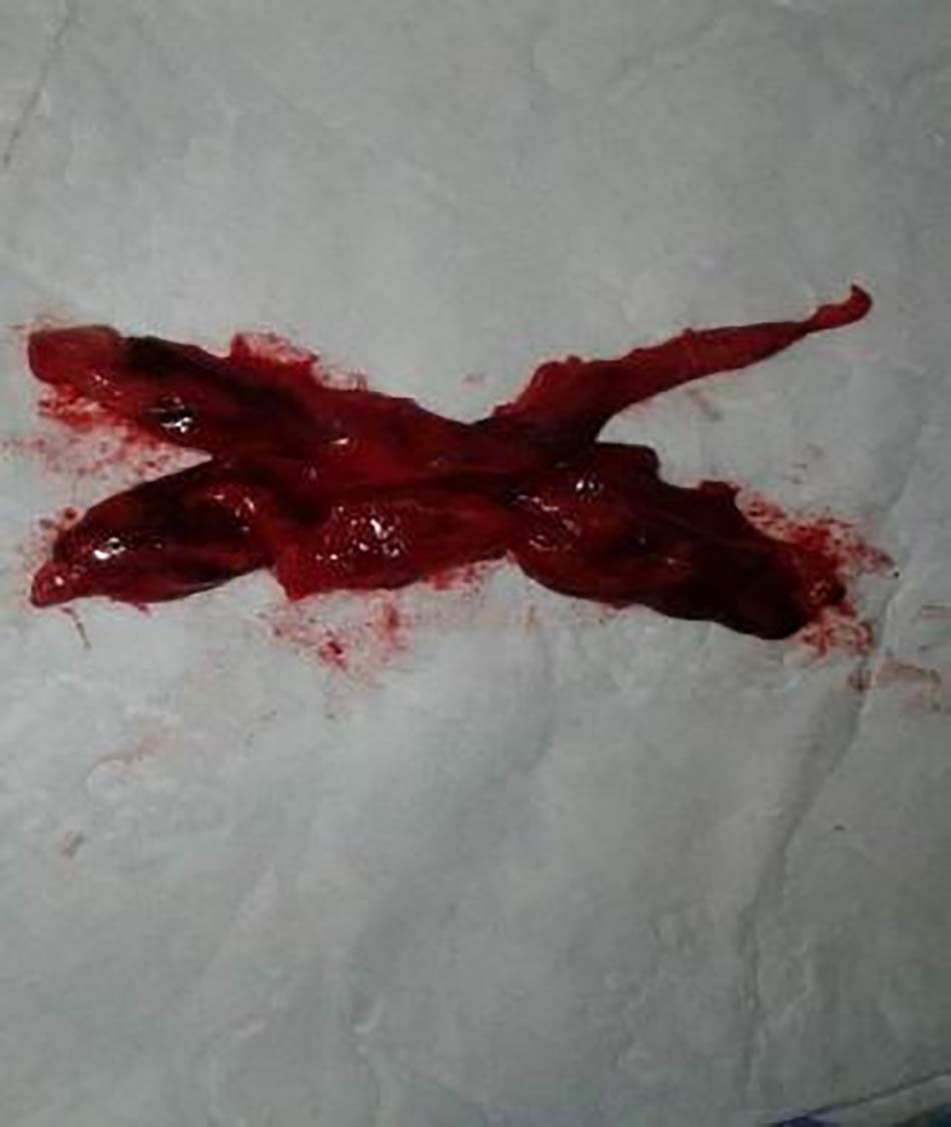
Resected thymoma case 1.

A 10-year-old male patient presented with muscle weakness, fatigue, loss of muscle power on repetitive movement by the end of the day, mild bulbar symptoms and respiratory muscle affection with Class IIIa on MGFA classification. Electromyography (EMG) showed decrements of the muscle action potential bilaterally in proximal lower limb more than distal Nerve conduction study (NCS) was normal with no evidence of L5-S1 root lesion. The chest computer tomography (CT) (Fig. 1) scan showed regressed homogenous thymic tissue with clear lung fields, no pleural or pericardial effusion, and no evidence of enlarged mediastinal or hilar lymph nodes. Heart and great vessels were normal. Mediastinoscopy biopsy and histopathology confirmed the diagnosis of thymoma. Surgical resection via open thymectomy showed invasive thymoma infiltrating the upper lobe (Fig. 2). Histology showed a cortical thymoma that represents a type B2 tumor according to WHO grade and the patient’s symptoms improved post-resection.

### Case 2

**Figure 3:**
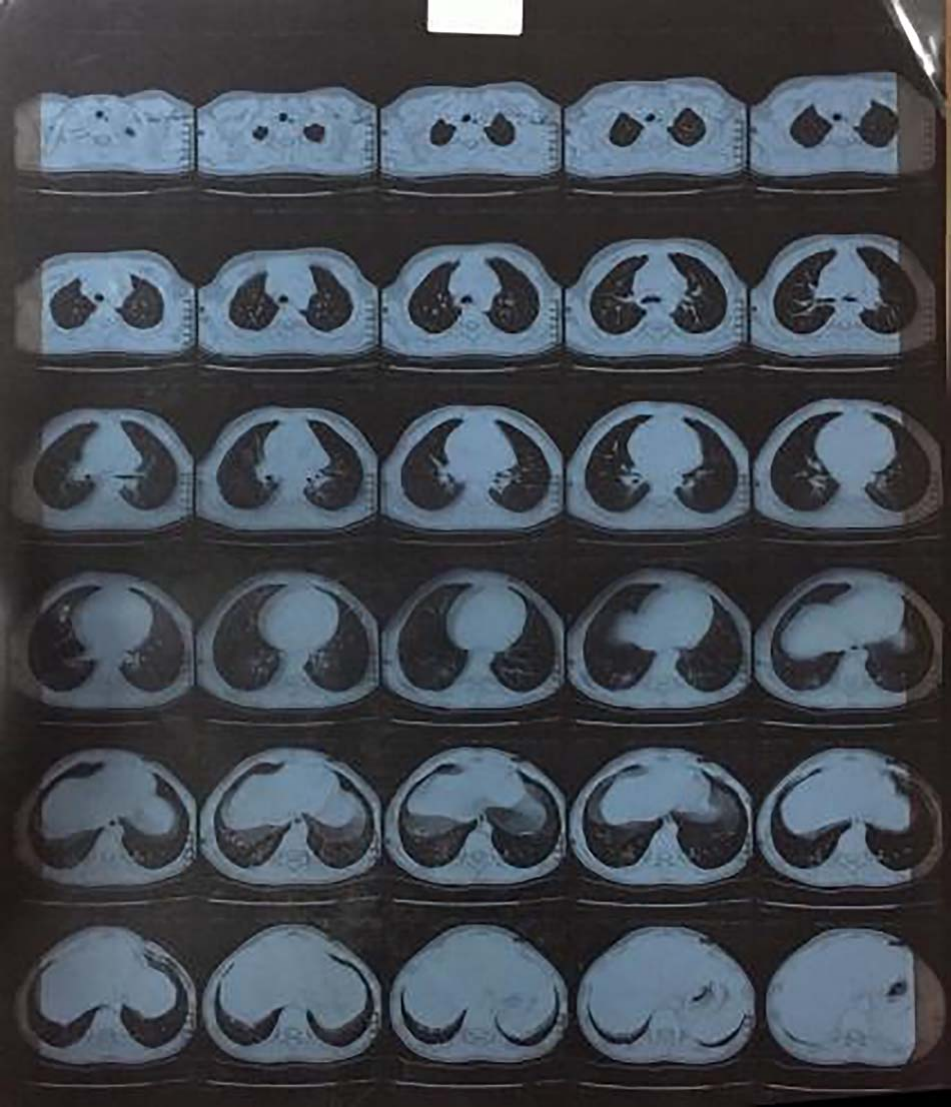
CT showing homogeneously hypo-echoic thymic mass seen in the retroperitoneal region at the level of the right hilum with enlarged lymph nodes.

**Figure 4:**
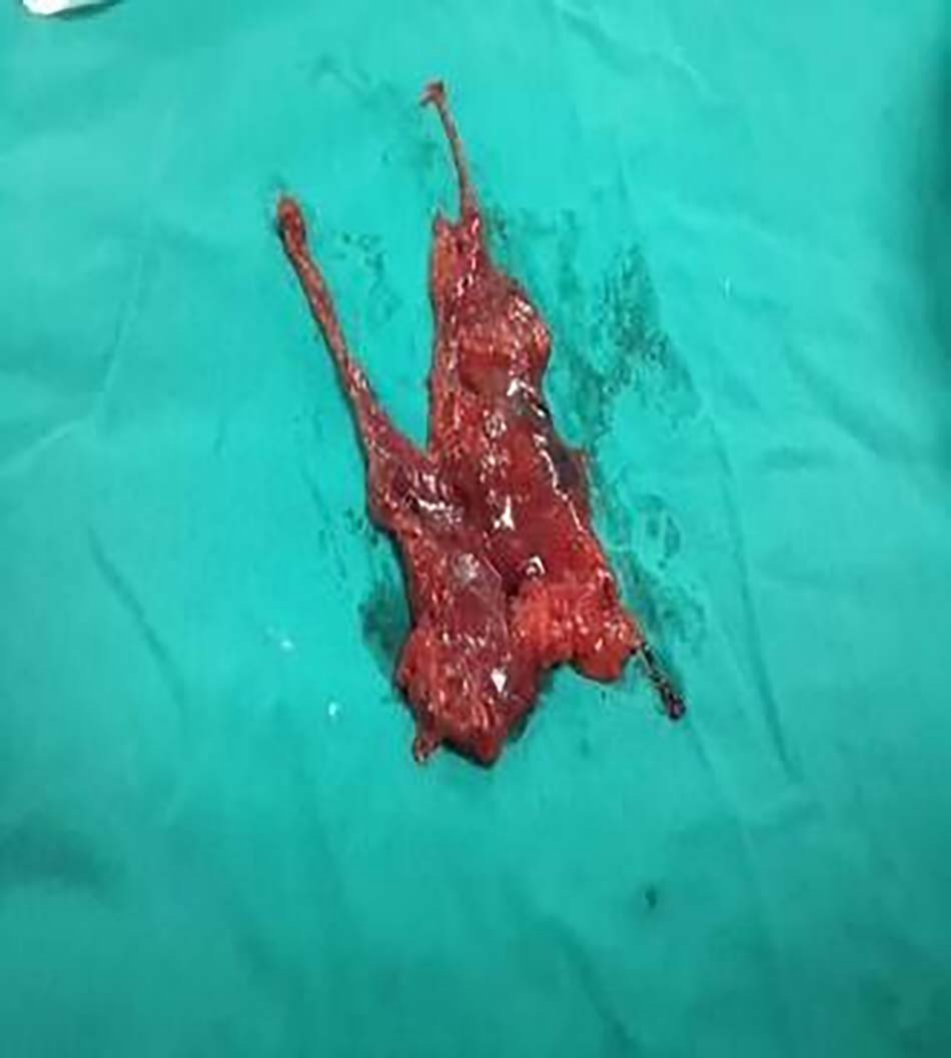
Resected thymoma case 2.

A 5-year-old presented with bilateral ptosis, which is more predominant in his left eye muscle weakness and inability to stand up by the end of the day. EMG & NCS suggested Myasthenic syndrome after repetitive nerve stimulation of the right median and tibial nerve before and after exercise. It showed 100% decremental response. Further analysis by a chest CT scan showed a well-defined homogeneously hypoechoic mass 3 × 2 cm seen in the retroperitoneal region at the level of the right hilum with an enlarged left thymic mass (Fig. 3). Open thymectomy (Fig. 4) was followed by thymectomy gross anatomy specimen measuring 12 × 10 × 2 cm, weighing 70 g, with a smooth glistening capsulated outer surface. The cut section was tan rubbery lobulated with areas of hemorrhage. Histopathology showed a hyperplastic lymphoid tissue with many Hassall’s corpuscles with no necrosis or nuclear anaplasia. There was no indication of malignancy in the sample. Post-surgical resection leads to resolution of myasthenic symptoms.

### Case 3

**Figure 5:**
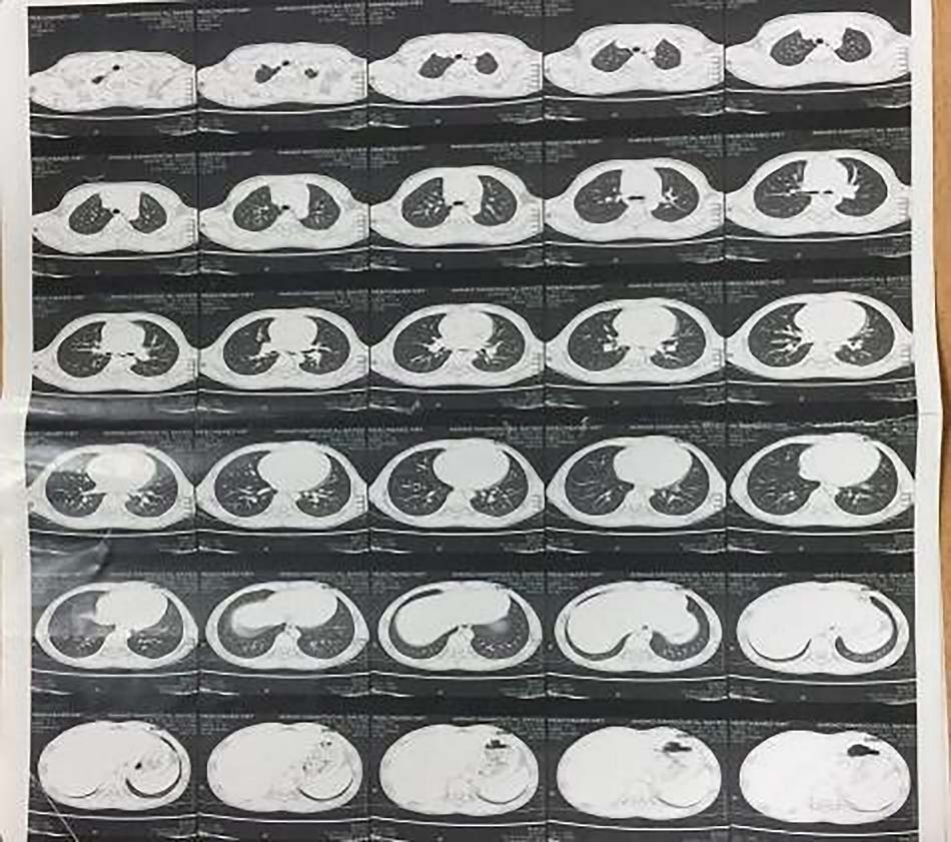
CT of slightly prominent thymus gland due to reactive hyperplasia and no significantly enlarged mediastinal lymph nodes.

**Figure 6:**
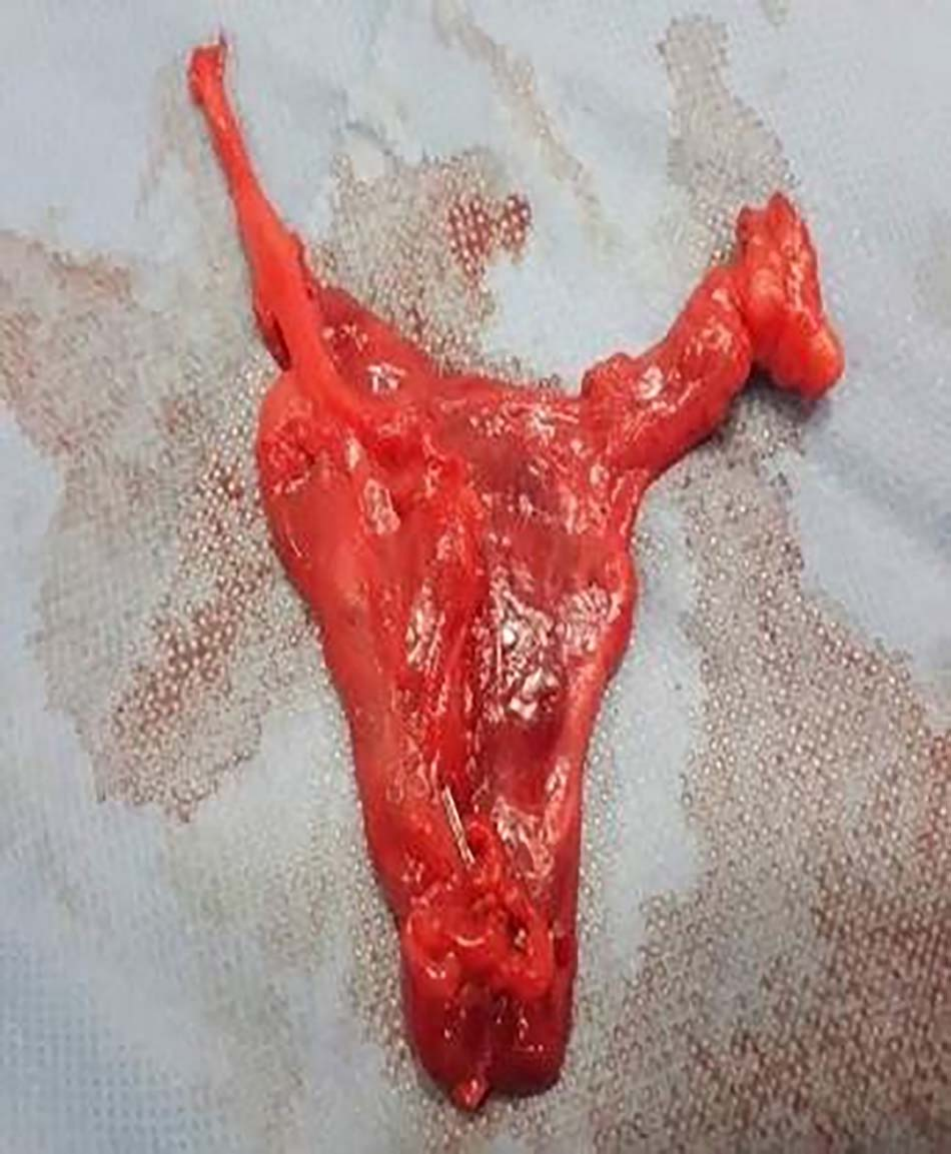
Resected thymoma case 3.

An 8-year-old-male patient presented for 1 year with bilateral ptosis and diplopia that improved with rest without bulbar symptoms, limb weakness or chest pain. On the examination, he presented with asymmetrical ptosis on repetitive movement. Both sensory and motor functions were unaffected and were graded Class I on the MGFA classification. EMG did not show any abnormalities. The AchR antibody titers were elevated at 9.0 nM (was positive). The NCS showed repetitive stimulation of facial nerve “orbicularis oculi” giving a positive decremental response. Histopathological analysis of the specimen showed thymic tissue with hyperplastic lymphoid tissue with many Hassel’s corpuscles. No necrosis or nucleus anaplasia was detected. Chest CT scan revealed slightly prominent thymus gland in the anterior mediastinum due to reactive hyperplasia and no significantly enlarged mediastinal lymph nodes (Fig. 5). The patient was placed on pyridostigmine and steroids and was scheduled for open thymectomy. Surgical resection was done (Fig. 6) and subsequently the patient’s symptoms resolved.

## DISCUSSION

Acquired myasthenia gravis is an autoimmune phenomenon that is linked to highly specific targeting of the anti-Ach antibodies that attacks the myoneural junction [1]. The classic motor symptoms of this disorder range from diplopia, ptosis and weakness of limbs on repetitive movements up to respiratory muscle affection and bulbar symptoms [2]. Nearly 15% of acquired myasthenia gravis cases are linked to thymomas [3]. Thymoma is a low-grade mediastinal epithelial tumor, which arises from the thymic tissue [4]. The association between myasthenia gravis and thymomas is a result of an immune response directed to epitopes on thymoma cells shared with the neuromuscular junction component; AChR antibodies are the main cause of muscle weakness in thymomatous MG. Additional non-AChR muscle autoantibodies reacting with striated muscle titin and ryanodine receptor (RyR) antigens are found in up to 95% of MG patients with thymoma [1]; however, autoantibodies titers is the mainstay of diagnosis and planning for the management protocol of thymomatous MG; in addition, these titers reflect the degree of the disease severity [3]. Although not reported on a wide scale, a rare subtype of this disease have been rarely reported known as the familial thymomatous MG [5, 6], which accounts for only 3–5% of thymomatous MG [6]. The diagnosis of this variant is based on a systematic approach beginning from the specific diagnosis of the myasthenic disorder by assessing the anti-AChR antibodies titers [1] and classifying its degree of MG via the MGFA classification (Table 1). This is followed by the thorough search or exclusion of an underlying thymic pathology by chest CT scan. Finding an anterior mediastinal mass would mandate a biopsy either through needle or mediastinoscopy [7] according to its accessibility. Histopathology will determine the grade of the tumor according to the WHO grading system (Table 2). Surgical intervention depends on the size and invasion of the thymic tumor; possible interventions include either video-assisted thoracoscopic approach (VATS) or open surgery [8]. However, open thymectomy is the surgical procedure of choice in children [9]. The endpoint for the diagnosis of this rare familial type can be confirmed by histopathology and further immunophenotypic assessment of the surface markers.

**Table 1:** WHO histopathological classification of thymic tumors

Type	Pathological classification	Prognosis
A	Medullary thymoma	Benign clinical course
	Spindle cell thymoma	
AB	Mixed thymoma	Moderately malignant clinical course
B1	Lymphocyte- rich thymoma	
	Lymphocytic thymoma	
	Predominantly cortical thymoma	
	Organoid thymoma	
B2	Cortical thymoma	
B3	Epithelial thymoma	
	Atypical thymoma	
	Squamoid thymoma	
	Well differentiated thymic carcinoma	
C	Thymic carcinoma	Highly malignant clinical course

**Table 2:** Grading system for myasthenia gravis diagnosis

Class	Clinical symptoms
I	Any ocular weakness
II	Mild weakness. May also have ocular muscle weakness of any severity
IIA	Predominantly affecting limb, axial muscles, or both. May also have lesser involvement of oropharyngeal, respiratory muscles or both
IIB	Predominantly affecting oropharyngeal, respiratory muscles, or both. May also have lesser or equal involvement of limb, axial muscles or both
III	Moderate weakness affecting other than ocular muscles. May also have ocular muscle weakness of any severity
IIIA	Predominantly affecting limb, axial muscles, or both. May have also lesser involvement of oropharyngeal, respiratory muscles or both
IIIB	Predominantly affecting oropharyngeal, respiratory muscles, or both. May also have lesser or equal involvement of limb, axial muscles or both
IV	Sever weakness affecting other than ocular muscles. May also have ocular muscle weakness of any severity
IVA	Predominantly affecting limb, axial muscles, or both. May also have lesser involvement of oropharyngeal, respiratory muscles or both
IVB	Predominantly affecting oropharyngeal, respiratory muscles, or both. May have lesser or equal involvement of limb, axial muscles or both
V	Defined by intubation, with or without mechanical ventilation, except when employed during routine post-operative management

In conclusion, familial thymomatous MG is a rare variant accounting for only 5% of overall thymomatous myasthenia gravis, which reflects the scarcity of the case throughout the world. The diagnosis of this disorder depends on many criteria. The most important one is the titer of anti-Ach antibodies, which is the most accurate tool for the diagnosis of myasthenia gravis. Such should steer the decision of thymectomy with complete resection of the mediastinal tumor either by open thymectomy or VATS. This depends on the patient case and age that will eventually lead to the resolution of the myasthenic symptoms.
